# Combined Analysis of Early CD4^+^ T Cell Counts and CMV Serostatus May Improve CMV Risk Assessment after Allogeneic Hematopoietic Cell Transplantation

**DOI:** 10.3390/cells10123318

**Published:** 2021-11-26

**Authors:** Saskia Leserer, Esteban Arrieta-Bolaños, Ulrike Buttkereit, Dietrich W. Beelen, Amin T. Turki

**Affiliations:** 1 Computational Hematology Lab, Department of Hematology and Stem Cell Transplantation, University Hospital Essen, 45122 Essen, Germany; saskia.leserer@uk-essen.de; 2 Department of Hematology and Stem Cell Transplantation, University Hospital Essen, 45122 Essen, Germany; ulrike.buttkereit@uk-essen.de (U.B.); dietrich.beelen@uk-essen.de (D.W.B.); 3 Institute for Experimental Cellular Therapy, University Hospital Essen, 45122 Essen, Germany; esteban.arrieta-bolanos@uk-essen.de; 4 German Cancer Consortium (DKTK), Partner Site Essen/Düsseldorf, 45147 Essen, Germany

**Keywords:** clustering, unsupervised learning, CMV risk assessment, CMV reactivation, CMV serostatus, CD4^+^ T helper cells, immune reconstitution, T cell reconstitution, R+ serostatus

## Abstract

The incidence and severity of viral complications after cellular therapy are highly variable. Recent publications describe relevant interactions between the human Cytomegalovirus (CMV) and host immunity in recipients of allogeneic hematopoietic cell transplantation (HCT). Although immune monitoring is routinely performed in HCT patients, validated cut-off levels correlating with transplant outcomes such as survival or CMV reactivation are mostly limited to day +100, which is later than the median time for CMV reactivation in the absence of medical prophylaxis. To address this gap in early risk assessment, we applied an unsupervised machine learning technique based on clustering of day +30 CD4^+^ helper T cell count data, and identified relevant cut-off levels within the diverse spectrum of early CD4^+^ reconstitution. These clusters were stratified for CMV recipient serostatus to identify early risk groups that predict clinical HCT outcome. Indeed, the new risk groups predicted subsequent clinical events such as NRM, OS, and high CMV peak titers better than the most established predictor, i.e., the positive CMV recipient serostatus (R+). More specifically, patients from the R+/low CD4^+^ subgroup strongly associated with high CMV peak titers and increased 3-year NRM (subdistribution hazard ratio (SHR) 10.1, 95% CI 1.38–73.8, *p* = 0.023), while patients from the R-/very high CD4^+^ subgroup showed comparable NRM risks (SHR 9.57, 95% CI 1.12–81.9, *p* = 0.039) without such an association. In short, our study established novel cut-off levels for early CD4^+^ T cells via unsupervised learning and supports the integration of host cellular immunity into clinical risk-assessment after HCT in the context of CMV reactivation.

## 1. Introduction

Cytomegalovirus (CMV) reactivation remains the most frequent viral complication after hematopoietic cell transplantation (HCT) [1]. While improved prophylaxis and pre-emptive therapy strategies have reduced CMV-associated morbidity, individual patients are still severely affected, and significant patient populations are exposed to relevant toxicity associated with CMV treatment [2]. Increased evidence of synergic processes between immune reconstitution and viral reactivation that influence clinical outcome after HCT has been reported in recent studies, focusing both on CMV biology and host immunity [3,4]. Recent publications have highlighted the importance of the absolute copy number of CMV viral loads, as a surrogate of CMV’s burden of disease [5,6,7] for clinical outcome, and associated lower early CD3^+^/CD4^+^ counts after HCT with the incidence of later high CMV peak titers [5]. Other studies showed interactions between treatment-resistant CMV reactivation requiring CMV-specific cytotoxic T cell therapy and the host’s endogenous CD4^+^ immunity, as revealed by baseline CD4^+^ counts [8]. Current screening and prevention practices after HCT insufficiently account for the impact of the host immune system, in its interaction with CMV. Besides in vivo T cell depletion or HCT from unrelated donors, the standard risk indicator of CMV viremia is still the CMV serostatus of recipient (R) and donor (D) [9,10], which are considered for HCT donor selection. Furthermore, R+ serostatus guides the use of CMV prophylaxis with letermovir [2], as CMV-R+ patients have been previously associated with worse clinical outcome after HCT [9,10]. Although predicting the overall incidence of CMV reactivation, previous data suggested that the R+ serostatus alone does not stratify patients for clinically relevant CMV peak titer risk groups [5]. Consequently, it remains challenging to differentiate patients at risk of progressing to high copy numbers with significant morbidity and hence requires pharmacological interventions for those who might be spared the toxicity of prolonged pre-emptive treatments.

Based on recent insights into the clinically relevant synergic processes between CMV and its host, the addition of early T cell immune reconstitution data to clinical risk assessment could improve CMV recipient (CMV-R) serostatus-based outcome prediction. Previous studies have defined relevant helper T cell cut-off levels, for improved clinical outcome, with absolute CD4^+^ counts of >50/µL at day+100 [11] and >200/µL at 12 months [12] after HCT. This association is based on the contribution of the helper T cell immunity in ensuring pathogen defence against CMV and other common viruses as well as its major role in the alloreactive eradication of residual malignant cells after HCT [13]. Despite the successful validation of these T helper cell cut-off levels in paediatric patients receiving T cell depletion with anti-thymocyte globulin (ATG) [14], in patients with graft-versus-host disease (GVHD) [15], and in HCT with different graft sources [16], their application in the context of CMV exposed patients is limited by the median onset of the first CMV reactivation episodes after HCT, which is as early as day+33 without prophylaxis [5]. We hypothesized that an unsupervised machine learning approach, namely a *k*-means data clustering, of CD4^+^ helper T cell counts at day+30 after HCT, combined with the R serostatus might identify early predictors of CMV-dependent clinical outcomes, and thus might improve the CMV-based risk assessment post-transplantation.

## 2. Materials and Methods

### 2.1. Patient Population

This retrospective study included a subset of 266 patients from a previously published cohort [5] with allogeneic HCT between January 2012 and December 2017, performed at the Department of Hematology and Stem Cell Transplantation of the West-German Cancer Center at University Hospital Essen. Based on our objectives, the initial study cohort of 705 patients [5] was filtered for patients with available flow cytometry data around day+30 after HCT. Follow ups were performed until the patients’ last clinical assessment (closing date on 4 June 2020), or death by any cause. Surviving patients were censored at maximum follow-up. HLA matching was evaluated at the 10/10 level, without considering HLA-DPB1. Mismatches between patients and donors were limited to one allele difference. Early supportive and follow-up care followed standard internal protocols and was identical for all patients. Immunosuppression after HCT was based on uniform pharmacological GVHD prophylaxis with calcineurin inhibitors, with 3 mg/kg body weight ciclosporin starting from day −1 before HCT combined with 15 mg/m^2^ methotrexate (MTX) on day +1 and 10 mg/m^2^ on days +3, +6 and +11 after HCT [17,18]. Inpatients were assessed three times per week for ciclosporin target blood levels (range, 150–250 ng/mL) and were orally substituted before discharge. Patients undergoing unrelated donor HCT (MUD or MMUD) received additional GVHD prophylaxis with polyvalent rabbit-anti-Jurkat-T-lymphocyte globulin (ATG, ATG Fresenius/Neovii, 97% of unrelated donor transplantations) at a dosage of 10 mg/kg or 20 mg/kg on days −4, −3 and −2 (cumulative dosages: 30 mg/kg or 60 mg/kg, respectively). Ex vivo T cell depletion was not applied.

### 2.2. Patient Assessment

All baseline data concerning patient-, donor- and HCT-characteristics and clinical outcomes were prospectively documented in electronic forms. Patient clinical characteristics and laboratory parameters were retrospectively analyzed. For inpatients, a daily clinical assessment was obtained, while subsequent outpatient follow-up was sequentially extended depending on clinical performance and transplant-associated complications. Overall survival (OS) was calculated from the day of transplantation to the maximum 3 years follow-up or death of any cause. The cumulative incidence of relapse was determined from the day of transplantation to the day of documented relapse or persistence of the original disease. For deceased patients without diagnosed relapse or persistency, non-relapse mortality (NRM) was calculated as the time from HCT to death. Relapse and NRM were considered as competing events. Acute graft-versus-host disease (aGVHD) was clinically assessed and classified according to the consensus criteria [19].

### 2.3. CMV Monitoring

Prior to HCT, both the recipient and donor were screened for CMV IgG antibodies. Molecular CMV detection after HCT was performed at the Institute for Virology at the University Hospital Essen as previously described [5]. In short, peripheral blood samples were screened for CMV by quantitative PCR (qPCR) twice weekly for inpatients and weekly for outpatients until day+100, and through extended intervals following this period. Between February 2012 and August 2013, the Artus CMV Real-time qPCR kit (Qiagen GmbH, Hilden, Germany) with a detection limit of 150 copies/mL was used; from August 2013 until the study’s end, monitoring was performed via the CMV Real-time qPCR from Abbot Molecular (Des Plaines, IL, USA) with a detection limit of 40 copies/mL. Data obtained from both kits were shown to be comparable [5]. The occurrence of clinically relevant CMV reactivation was defined as >500 copies/mL. Patients with CMV reactivation received a pre-emptive therapy with ganciclovir twice daily at 5 mg/kg of patient body weight for 14 days. In case of non-response to first-line treatment, foscarnet or cidofovir were applied according to the physician’s choice and the toxicity profile.

### 2.4. Flow Cytometry

Flow cytometry analysis was performed at the BMT Flow Cytometry Laboratory at University Hospital Essen using whole blood samples of patients from around day +30 as previously reported [5]. Helper T cells (CD3^+^/CD4^+^) were gated on the CD45^+^ lymphocyte gate. Absolute helper T cell counts were calculated utilizing absolute lymphocyte counts and the percentages of this subset.

### 2.5. Unsupervised Clustering of CD4^+^ Helper T Cells

In order to identify homogenous and unbiased patient subgroups, absolute helper T cell counts, obtained by flow cytometry, from 266 patients were scaled as Log_10_ values and grouped using an unsupervised *k*-means clustering method based on the Euclidean distance from each cluster’s center. We limited the number of possible clusters to six. Clusters with less than 25 patients were combined with neighboring clusters, yielding to the final four CD4^+^ T cell clusters. Calculations were performed using Statistical Package for the Social Science (SPSS 23.0, SPSS Inc., Chicago, IL, USA; IBM, Armonk, NY, USA) following the manufacturer’s instructions (see https://www.ibm.com/docs/en/spss-statistics/SaaS?topic=features-means-cluster-analysis, accessed on 16 September 2020). In the second step, CD4^+^ clusters were stratified for the R serostatus (R+/R−) resulting in 8 patient subgroups, which were subsequently correlated with clinical outcomes and CMV peak titers.

### 2.6. Statistical Analysis

Patient percentages in the combined groups of R serostatus and CD4^+^ clusters with respect to published CMV peak titers [5] were calculated and illustrated using GraphPad Prism (Version 9.0.0, GraphPad Software Inc., San Diego, CA, USA). The 3-year OS was calculated using the Kaplan–Meier method [20]. Hazard ratios for OS were calculated using the Cox proportional hazards model [21]. Differences in survival outcomes between groups were compared using the log-rank test. NRM and cumulative relapse incidence were analyzed using the Fine and Gray competing risks regression [22], comparing cumulative incidence functions in different groups with the Gray test. This model estimates the effect of covariates on the subdistribution of a specific event in the competing risks setting, producing subdistribution hazards (SHR). *p*-values < 0.05 were considered statistically significant. All outcome analyses were performed using R software [23] (version 4.0.4, R Development Core Team, Vienna, Austria) with the packages survival [24], survminer [25], cmprsk [26] and ggplot2 [27].

## 3. Results

### 3.1. Patient Characteristics

A total of 266 patients with HCT between January 2012–December 2017 were included in this retrospective analysis and independently analyzed, with a focus on the identification of clinically relevant predictors and the interaction between CMV serostatus and early host immune reconstitution. This patient subset was comparable to a previously published, larger cohort regarding the proportion of patients with CMV reactivation > 500 copies/mL (50% vs. 50% [5]) and the median time taken for first CMV reactivation (day + 32 vs. day + 33 [5]). Acute myeloid leukemia was the predominant disease. Baseline characteristics in the cohort are detailed in Table 1.

**Figure 1:**
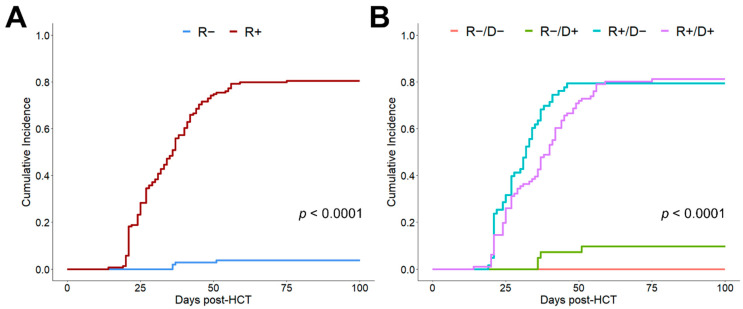
Cumulative incidence of CMV reactivation depending on recipient/donor CMV serostatus. (**A**) Cumulative incidence of 100-day CMV reactivation depending on the recipient (R) serostatus. (**B**) Cumulative incidence of 100-day CMV reactivation depending on recipient (R) and donor (D) CMV serostatus.

An analysis regarding the influence of the CMV serostatus of recipient and donor on the incidence of CMV reactivation until d + 100 confirmed this event’s dependency on CMV R+ seropositivity (Figure 1A, *p* < 0.0001). Only in CMV seronegative recipients did the donor serostatus significantly influence the risk of CMV reactivation (Figure 1B).

### 3.2. Improved Outcome Prognosis Based on K-Means CD4^+^ Helper T Cell Clusters

**Figure 2:**
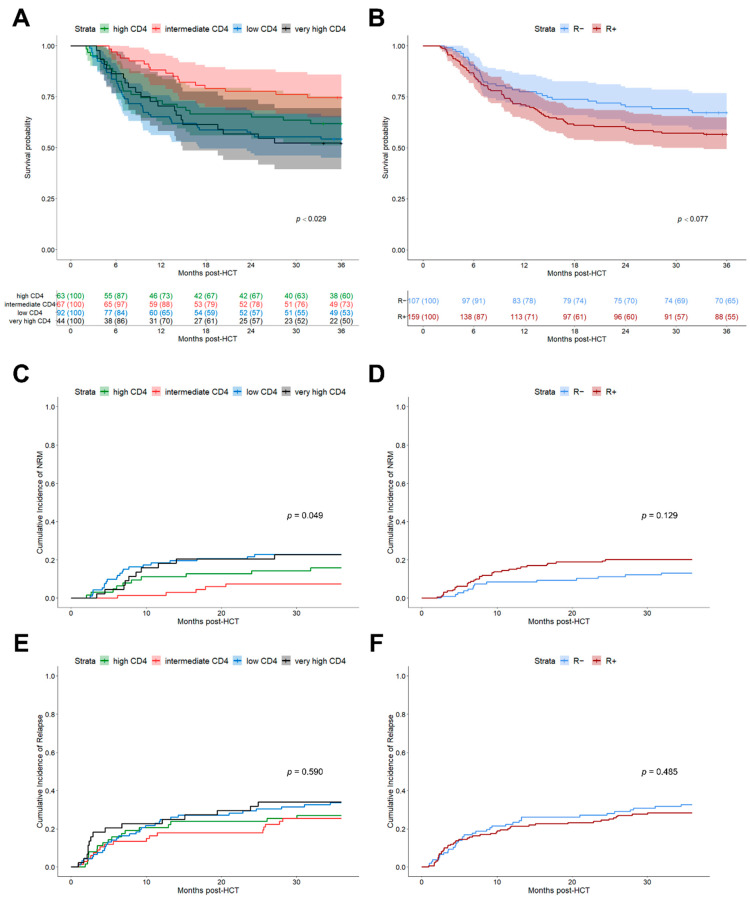
Clinical outcomes after HCT depending on CMV risk model. (**A**) Kaplan–Meier analysis of OS stratified for 4 *k*-means clusters of CD4^+^ helper T cells at day +30 after HCT. Clusters: Low CD4^+^ (0–39 cells/µL, *n* = 92, blue), intermediate CD4^+^ (40–105 cells/µL, *n* = 67, red), high CD4^+^ (106–260 cells/µL, *n* = 63, green), and very high CD4^+^ (≥261 cells/µL, *n* = 44, black). Line indicates median with 95% confidence interval (CI) shaded. Comparison of strata via log-rank test. (**B**) Kaplan–Meier analysis of OS stratified for CMV recipient serostatus (R+, *n* = 159, red; R−, *n* = 107, blue). Median (line) with 95% CI shaded. Comparison of strata via log-rank test. (**C**,**D**) Cumulative incidence function of 3-year NRM depending on: (**C**) *k*-means CD4^+^ helper T cell clusters and (**D**) recipient serostatus. (**E**,**F**) Cumulative incidence function of 3-year relapse depending on: (**E**) *k*-means CD4^+^ helper T cell clusters and (**F**) recipient serostatus.

*K*-means clustering of Log_10_ CD4^+^ helper T cell counts at day +30 resulted in the identification of four clusters with the following cut-off levels: low (0–39 cells/µL, *n* = 92), intermediate (40–105 cells/µL, *n* = 67), high (106–260 cells/µL, *n* = 63) and very high (≥261 cells/µL, *n* = 44). Interestingly, these *k*-means clustered early CD4^+^ helper T cell subgroups differed significantly for clinical outcome after HCT, with better stratification than the recipient CMV serostatus alone (3-year OS, *p* = 0.029 vs. *p* = 0.077, Figure 2A,B). The observed numerically lower OS, increased NRM and comparable relapse incidences of R+ patients compared to R− ones, (Figure 2D,F) were consistent with the results from large registry studies [9,28]. Besides superior OS stratification, helper T cell clusters also led to better differentiation regarding NRM (*p* = 0.049, Figure 2C), as patients with low (*n* = 92) and very high (*n* = 44) CD4^+^ T cell counts at day +30 after HCT had significantly higher NRM, consistent with reduced OS. Neither helper T cell clusters (Figure 2E) nor CMV R+ serostatus (Figure 2F) subgroups differentiate significantly for relapse. Early CD4^+^ T cell counts did not correlate with the severity of aGVHD (Table 2).

### 3.3. Stratification of Helper T Cell Clusters by Recipient Serostatus Further Improves Clinical Prognosis

**Figure 3:**
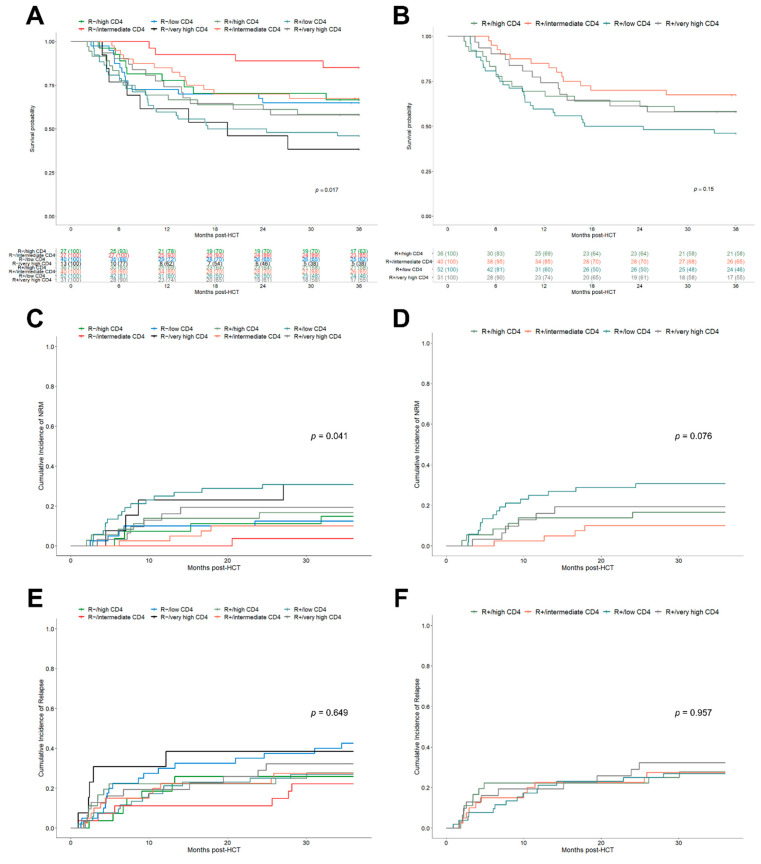
Clinical outcomes after HCT depending on the combined R serostatus/helper T cell cluster model. (**A**) Kaplan–Meier analysis of OS stratified for combined R serostatus/CD4^+^ helper T cell subgroups. R+/low CD4^+^ (turquoise), R+/intermediate CD4^+^ (orange), R+/high CD4^+^ (dark green), R+/very high CD4^+^ (grey) and R−/low CD4^+^ (blue), R−/intermediate CD4^+^ (red), R−/high CD4^+^ (green), R−/very high CD4^+^ (black). (**B**) Kaplan–Meier analysis of OS stratified for combined R+/CD4^+^ helper T cell subgroups. Colors as in (**A**). (**C**,**D**) Cumulative incidence function of 3-year NRM depending on: (**C**) R serostatus/CD4^+^ helper T cell subgroups and (**D**) R+/CD4^+^ helper T cell subgroups. (**E**,**F**) Cumulative incidence function of 3-year relapse depending on: (**E**) R serostatus/CD4^+^ helper T cell subgroups and (**F**) R+/CD4^+^ helper T cell subgroups.

After demonstrating a sufficient differentiation of patient outcomes using the *k*-means clustered early helper T cell subgroups, we further attempted to leverage those groups with the existing risk factor of CMV recipient seropositivity. Indeed, the stratification of helper T cell subgroups for the R−/R+ serostatus further improved the significance level with respect to OS (Figure 3A, *p* = 0.017), showing a more precise distinction of clinical outcomes based on the combined CMV-R serostatus/helper T cell cluster subgroups.

The observed 3-year OS ranged from 85.2% (95% CI, 72.8–99.7) for the R−/intermediate CD4^+^ subgroup to 46.2% (95% CI, 34.4–61.9) for the R+/low CD4^+^ subgroup. The R serostatus/helper T cell subgroups were also found to be associated with significant differences in NRM (Figure 3C, *p* = 0.041) but not for relapse (Figure 3E). These results were also confirmed by the Fine and Gray competing risk regression (Table 3). Interestingly, both patients of the R−/low CD4^+^ and R−/very high CD4^+^ subgroups had the highest relapse rates (42.5%, 95% CI 29.6–60.9 and 38.5%, 95% CI 19.3–76.5, respectively) presumably resulting in lower OS. After limiting the evaluation to patients with detected CMV reactivation (*n* = 133), a Cox regression analysis was performed, which also corroborated the above-reported results. In particular, the R+/low CD4^+^ subgroup was significantly associated with reduced OS (HR 2.88, 95% CI 1.35–6.14, *p* = 0.006) translating into a 3-year OS of 40.9% (95% CI 28.7–58.4). However, the confirmation of results for the R−/very high CD4^+^ subgroup in CMV reactivation patients was limited due to low patient numbers in this subgroup. Focusing on the subset of seropositive recipients (*n* = 159), helper T cell clusters identified separate outcome groups, showing superior, although not statistically significant, results for patients with intermediate CD4^+^ levels in 3-year OS (Figure 3B, *p* = 0.15), lower NRM (Figure 3D, *p* = 0.076) and a comparable relapse incidence (Figure 3F).

### 3.4. Combined R Serostatus/Helper T Cell Clusters Associate with Clinically Relevant CMV Reactivation

**Figure 4:**
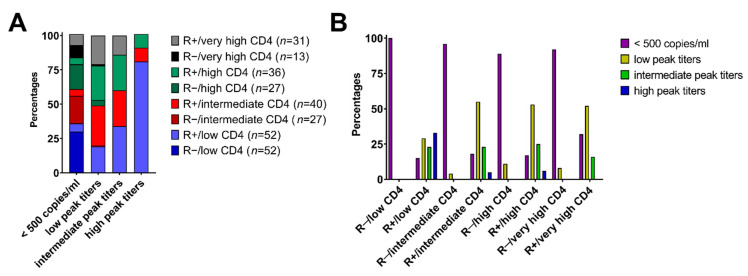
Association of the combined R serostatus/helper T cell cluster subgroups with CMV peak titers. (**A**) Proportion (%) of combined R serostatus/CD4^+^ cohorts with corresponding CMV peak titers (cut-off values: negative (<500 copies/mL), low peak titers (500–20,000 copies/mL), intermediate peak titers (20,000–100,000 copies/mL) and high (>100,000 copies/mL), respectively). (**B**) Proportion of patients (%) of different CMV peak titers per combined R serostatus/CD4^+^ risk group.

Based on recent data underlining the clinical relevance of CMV peak titers [5] we proceeded to evaluate its association with our combined R serostatus/helper T cell cluster model. Interestingly, R+ patients progressed to distinct CMV peak titers depending on their CD4^+^ helper T cell levels at day+30 after HCT (*p* = 0.0003). Patients of the R+/low CD4 subgroup had the highest probability to progress to high CMV peak titers (Figure 4A). However, patients with a helper T cell reconstitution to intermediate levels (40–105 cells/µL) were less likely (6%) to experience high CMV peak titers, and the likelihood further declined with increasing early CD4^+^ levels (Figure 4B). Importantly, patients from the very high CD4^+^ clusters appeared to be protected against detrimental CMV reactivations with high peak titers irrespective of their serostatus (Figure 4A). R− patients only had a very low probability of CMV reactivation with low peak titers, independent of their early CD4^+^ reconstitution levels (Figure 4A,B).

## 4. Discussion

The identification of appropriate predictors for CMV-associated risk beyond the CMV serostatus using cellular markers of host immunity is of great interest, particularly in patients with a high probability for CMV reactivation, in order to better understand the impact of early cellular immune recovery on CMV-dependent outcome. However, previous attempts analyzing such cellular markers have been either limited to later time points around month+3 after HCT [29], or focused on the area under the curve within the first 3 months [11]. These approaches do not fully reflect host immunity at the median time of CMV reactivation, which is usually around day+30 after HCT in patients without medical CMV prophylaxis [5,9,30]. As seropositive patients are eligible for letermovir prophylaxis, which prevents CMV reactivation and delays its onset in some patients [2], early predictors may further improve risk stratification in this population, leading to the identification of subsets of high-risk CMV-R+ patients. Here, we investigated the use of unsupervised *k*-means clustering for the detection of differential patterns of early helper T cell immune reconstitution. In this analysis, four distinct patient subgroups were identified based on their differential levels of helper T cell recovery. These subgroups were partially associated with increased NRM and subsequently, reduced OS, leading to a better outcome differentiation than the recipient CMV serostatus after HCT. The combination of both CD4^+^ reconstitution and R serostatus further improved the clinical prognosis and was shown to be associates with clinically relevant CMV peak titers [5]. Specifically, the R+/low CD4^+^ subgroup correlated with high peak titer CMV reactivations, which have been previously associated with lower OS and increased NRM [5], whereas R− patients predominantly had CMV viremia < 500 copies/mL. The final model of combined CD4^+^ T cell clusters and recipient seropositivity established a new approach for the identification of high-risk CMV-R+ patients by integrating parameters of host immune reconstitution in its interaction with CMV. Indeed, CMV prophylaxis with letermovir is crucial in the identified high-risk populations with both CMV-R+ serostatus and impaired CD4^+^ T cell reconstitution. Theoretically, the prolongation of CMV prophylaxis in patients with poor immune reconstitution may be considered, whereas prophylaxis might be hypothetically discontinued earlier in patients with an adequate CD4^+^ T cell reconstitution. However, these hypotheses will require further investigation in future studies.

Recently, rapid and sufficient helper T cell recovery has been associated with a decreased incidence of viral reactivations [31,32] and increased OS [11,29,32,33] after HCT. Data regarding its influence on relapse remain controversial [14,29,32,34,35]. However, other studies have challenged the beneficial effects on OS by showing a higher mortality rate in patients with peak CD4^+^ levels within 3 months of HCT [11]. Given this background, our data, providing four distinct early CD4^+^ clusters with different clinical outcomes, instead of one specific cut-off value, might explain the discrepancy between studies concerning the association of T cell recovery to OS. Our analyses support, on the one hand, the beneficial effect of a sufficient helper T cell reconstitution, for example in the intermediate CD4^+^ subgroup (40–105 cells/µL), but on the other hand, also provide evidence for a negative association of very high CD4^+^ levels (≥261 cells/µL) with OS. The latter might either relate to rapid peripheral expansion of CD4^+^ T cells due to aGVHD or viral reactivation events triggering CD4^+^ cell recovery [36,37], which has not been evaluated in this study. Furthermore, a very delayed early reconstitution of helper T cells (0–39 cells/µL) at d+ 30 after HCT was previously related to in vivo T cell depletion protocols with, e.g., ATG [38]. Although our cohort included a high proportion of patients with ATG (*n* = 189, 71%), this was not considered in the final CMV risk model. This limitation may be addressed in future studies of a larger scale. Beyond cellular immunity, as shown by our data, CMV may also be controlled by potent antibody responses, as previously revealed in an HCT mouse model [39]. However, the strain-specificity of such responses appears to be critical as the efficacy of preventing CMV with polyclonal intravenous immunoglobulins was limited in past studies [40]. In this context, CD4^+^ T cell immunity could also be indirectly involved in this process via its contribution to antibody production by B cells [41,42]. Here, insufficient CD4^+^ T cell levels might consequently lead to an impaired production of immunoglobulins against various pathogens, increasing the risk of post-HCT infections, including CMV reactivation.

Our data support the influence of the recipient CMV serostatus on CMV reactivation and other clinical outcomes as was demonstrated in previous analyses, showing an increase in NRM for seropositive recipients [9,28,43]. However, our data also suggest that this impact is further dependent on the level of cellular recovery. This was not only shown by differences in clinical outcomes in the R/CD4^+^ cluster subgroups but, additionally, through association with clinically relevant CMV peak titers [5,6]. The final and combined CMV-recipient serostatus/CD4^+^ T cell clusters improved the assignment of patients into a specific peak titer subgroup compared with the CMV serostatus alone, which might be clinically useful for the early identification of high-risk CMV seropositive HCT recipients. In particular, CMV-R+ patients with a low CD4^+^ count at month 1 after HCT are found to be at increased risks of high peak titer CMV reactivations and high NRM. Conversely, CMV-R− individuals experience CMV reactivations <500 copies/mL, irrespective of their cellular reconstitution. Finally, the low ratio of CMV reactivations in R− with D+ was previously associated with low or intermediate CMV peak titers showing either comparable outcomes to patients without reactivation or a reduced relapse ratio [5], further illustrating the complex interactions between these variables and clinical outcome.

The advantage of *k*-means clustering is its ability to provide more than two distinct subgroups for analysis, which is not achievable by, e.g., receiver operating characteristic (ROC) comparisons of pre-defined cut-off values with the best proportion of maximal specificity and sensitivity [44]. Given the presence of four subgroups in early cellular reconstitution after HCT, we were able to find evidence that could explain the controversial data regarding the association to OS in previous studies. Similar to the examination of CMV reactivation [5], not only one specific cut-off value appears to be of clinical relevance but the differentiation into several clusters might provide clarity for the overall clinical picture and may be more suited for the complexity of these interactions than a dichotomous analysis. However, this study has some limitations due to its sample size, retrospective character and missing functional immune assays. The number of patients receiving BM grafts, associated with a slower reconstitution of CD4^+^ T cells than PBSC grafts [45] was very small. Hence, this model is not necessarily applicable for such a setting. The integration of the developed risk model using the recipient serostatus and helper T cell clusters in clinical practice, especially as a decision-support system for, e.g., sustained CMV prophylaxis with letermovir, would require prospective evaluation in a clinical trial.

In conclusion, our results support the integration of host cellular immunity in the assessment of early CMV associated risks after HCT. Hence, the combination of the CMV recipient serostatus and early helper T cell counts at day+30 may practically improve early CMV-dependent risk assessment in HCT recipients. Further studies to prove this hypothesis in larger cohorts are warranted.

## Figures and Tables

**Table 1:** Patient baseline characteristics.

Characteristics	*n*	%
Total enrolled and treated	266	100
Median age at transplantation (range)	53	(18–76)
Female sex	115	43
**Disease**		
Acute myeloid leukemia	117	44
Myelodysplastic syndrome	21	8
Acute lymphoblastic leukemia	29	11
Chronic myeloid leukemia	12	5
Chronic lymphocytic leukemia	5	2
Chronic myelomonocytic leukemia	4	2
Non-Hodgkin lymphoma	30	11
Hodgkin lymphoma	5	2
Multiple myeloma	17	6
Myeloproliferative disorders	18	7
Other hematologic disorders	8	3
**Graft source**		
PBSC	254	95
BM	12	5
**Donor Type**		
MRD	70	26
MMRD	2	1
MUD	153	58
MMUD	41	15
**Recipient/Donor sex constellation**		
Female/Female	62	23
Male/Male	115	43
Female/Male	53	20
Male/Female	36	14
***CMV Serostatus***		
R+/D−	63	24
R+/D+	96	36
R−/D+	41	15
R−/D−	66	25
***Conditioning***		
MAC	110	41
RIC	156	59
TBI	101	38
ATG	189	71

Abbreviations: ATG, anti-T-lymphocyte globulin; BM, bone marrow; D, donor; MAC, myeloablative conditioning; MMRD, mismatched related donor; MMUD, mismatched unrelated donor; MRD; matched related donor; MUD, matched unrelated donor; PBSC, peripheral blood stem cells; R, recipient; RIC, reduced intensity conditioning; TBI, total body irradiation.

**Table 2:** Acute GVHD events in the CD4^+^ T cell clusters.

	Low CD4^+^ *n* (%)	Intermediate CD4^+^ *n* (%)	High CD4^+^ *n* (%)	Very High CD4^+^ *n* (%)	*p*
No aGVHD	10 (11)	11 (16)	7 (11)	11 (25)	0.132
aGVHD	82 (89)	56 (84)	56 (89)	33 (75)	
No aGVHD	10 (11)	11 (16)	7 (11)	11 (25)	0.341
Grade I	32 (35)	24 (36)	28 (44)	15 (34)	
Grade II	40 (43)	27 (40)	24 (38)	16 (36)	
Grade III	7 (8)	5 (7)	1 (2)	1 (2)	
Grade IV	3 (3)	0 (0)	3 (5)	1 (2)	

Differences of aGVHD in the CD4^+^ helper T cell clusters were tested using the Chi-square test.

**Table 3:** Fine and Gray competing risks regression.

Outcome	*n* (%)	Competing Risk Regression		
		SHR	95% CI	*p*
**Relapse ^‡^**				
R−/intermediate CD4	27 (10)	—	—	—
R−/low CD4	40 (15)	2.18	0.89–5.38	0.090
R+/low CD4	52 (20)	1.27	0.50–3.23	0.620
R+/intermediate CD4	40 (15)	1.32	0.50–3.52	0.570
R−/high CD4	27 (10)	1.21	0.42–3.51	0.720
R+/high CD4	36 (14)	1.38	0.50–3.76	0.530
R−/very high CD4	13 (5)	2.20	0.64–7.57	0.210
R+/very high CD4	31 (12)	1.60	0.59–4.31	0.360
**NRM ^‡^**				
R−/intermediate CD4	27 (10)	—	—	—
R−/low CD4	40 (15)	3.64	0.44–30.40	0.230
**R+/low CD4**	52 (20)	**10.1**	**1.38–73.80**	**0.023**
R+/intermediate CD4	40 (15)	2.78	0.32–23.90	0.350
R−/high CD4	27 (10)	4.22	0.49–36.40	0.190
R+/high CD4	36 (14)	4.97	0.61–40.20	0.130
**R−/very high CD4**	13 (5)	**9.57**	**1.12–81.90**	**0.039**
R+/very high CD4	31 (12)	5.71	0.71–45.80	0.100

Abbreviations: SHR, subdistribution hazard ratio; —, reference group; 95% CI, 95% confidence interval. ^‡^ Relapse and NRM were considered as competing events.

## Data Availability

On reasonable request, data is available from the corresponding author in accordance with ethical restrictions: amin.turki@uk-essen.de.
